# The Effects of Bipolar Cancellation Phenomenon on Nano-Electrochemotherapy of Melanoma Tumors: In Vitro and In Vivo Pilot

**DOI:** 10.3390/ijms25179338

**Published:** 2024-08-28

**Authors:** Eglė Mickevičiūtė, Eivina Radzevičiūtė-Valčiukė, Veronika Malyško-Ptašinskė, Paulina Malakauskaitė, Barbora Lekešytė, Nina Rembialkowska, Julita Kulbacka, Joanna Tunikowska, Jurij Novickij, Vitalij Novickij

**Affiliations:** 1 Department of Immunology and Bioelectrochemistry, State Research Institute Centre for Innovative Medicine, LT-08406 Vilnius, Lithuania; egle.mickeviciute@imcentras.lt (E.M.); eivina.radzeviciute@imcentras.lt (E.R.-V.); paulina.malakauskaite@imcentras.lt (P.M.); barbora.lekesyte@imcentras.lt (B.L.); julita.kulbacka@umw.edu.pl (J.K.); 2 Faculty of Electronics, Vilnius Gediminas Technical University, LT-10223 Vilnius, Lithuania; veronika.malysko-ptasinske@vilniustech.lt (V.M.-P.); jurij.novickij@vilniustech.lt (J.N.); 3 Department of Molecular and Cellular Biology, Wroclaw Medical University, 50-367 Wroclaw, Poland; nina.rembialkowska@umw.edu.pl; 4 Faculty of Veterinary Medicine, Wroclaw University of Environmental and Life Sciences, 50-375 Wroclaw, Poland; joanna.paczuska@upwr.edu.pl

**Keywords:** bipolar pulse, cancellation effect, high frequency, calcium electrochemotherapy, tumors

## Abstract

The phenomenon known as bipolar cancellation is observed when biphasic nanosecond electric field pulses are used, which results in reduced electroporation efficiency when compared to unipolar pulses of the same parameters. Basically, the negative phase of the bipolar pulse diminishes the effect of the positive phase. Our study aimed to investigate how bipolar cancellation affects Ca^2+^ electrochemotherapy and cellular response under varying electric field intensities and pulse durations (3–7 kV/cm, 100, 300, and 500 ns bipolar 1 MHz repetition frequency pulse bursts, n = 100). As a reference, standard microsecond range parametric protocols were used (100 µs × 8 pulses). We have shown that the cancellation effect is extremely strong when the pulses are closely spaced (1 MHz frequency), which results in a lack of cell membrane permeabilization and consequent failure of electrochemotherapy in vitro. To validate the observations, we have performed a pilot in vivo study where we compared the efficacy of monophasic (5 kV/cm × ↑500 ns × 100) and biphasic sequences (5 kV/cm × ↑500 ns + ↓500 ns × 100) delivered at 1 MHz frequency in the context of Ca^2+^ electrochemotherapy (*B16-F10* cell line, *C57BL/6* mice, n = 24). Mice treated with bipolar pulses did not exhibit prolonged survival when compared to the untreated control (tumor-bearing mice); therefore, the bipolar cancellation phenomenon was also occurrent in vivo, significantly impairing electrochemotherapy. At the same time, the efficacy of monophasic nanosecond pulses was comparable to 1.4 kV/cm × 100 µs × 8 pulses sequence, resulting in tumor reduction following the treatment and prolonged survival of the animals.

## 1. Introduction

The electric potential difference between the inner and outer sides of the cell plasma membrane (resting transmembrane voltage) is regulated by a system of ion pumps and channels in the cell membrane [1]. Exposure of biological cell lipid plasma membranes to external intense pulsed electric fields leads to additional polarization and an increase in the transmembrane voltage, which after a certain threshold results in the formation of hydrophilic pores due to the reorganization of lipids, a phenomenon known as electroporation (https://www.sciencedirect.com/topics/neuroscience/electroporation (accessed on: 14 March 2024)). This phenomenon exhibits itself in two forms: reversible electroporation, in which nano-pores are transient and cell membrane integrity is restored, and irreversible electroporation (IRE), in which permeabilization disrupts cellular homeostasis, which leads to cell death. Reversible electroporation (RE) is typically employed for targeted molecular delivery (drugs, genes, etc.), while IRE is predominantly used for tissue ablation in the biomedical context. Thus, both modalities have good applicability for the treatment of cancer. Electroporation efficacy and the modality of the effect (RE or IRE) depend on the specific parameters of the pulsed electric field, given its polarization-dependent nature [2,3], i.e., electric field amplitude, pulse number and duration, pulse shape, and other waveform characteristics.

The combination of reversible electroporation with cytotoxic drugs is known as electrochemotherapy (ECT) (https://www.sciencedirect.com/topics/medicine-and-dentistry/electrochemotherapy (accessed on: 14 March 2024))), when pulsed electric field (PEF)-mediated drug delivery significantly boosts the efficacy of chemotherapeutic drugs, leading to a more effective tumor eradication compared to chemotherapy [4]. This influx of drugs is facilitated by the pores formed during electroporation, enabling the use of lower doses of cytotoxic agents [5]. ECT is widely used in head and neck cancer [6], as well as liver [7], skin melanoma [8,9], and pancreatic cancer [10]. Recently, a new modality of electrochemotherapy was proposed known as calcium electrochemotherapy or calcium electroporation. Increased intracellular Ca^2+^ concentration accommodated by PEF causes ATP depletion, eventually causing cell apoptosis or necrosis processes [11]; thus, it can be used for the treatment of tumors [12]. In a clinical ECT setting, eight square-wave pulses of 100 μs delivered at a frequency of 1 Hz or 5 kHz are usually used, known as ESOPE (European Standard Operating Procedures for Electrochemotherapy) [13]. While ECT is considered a safe and efficient methodology, new parametric protocols are constantly being researched to improve the technique further. One of the biggest challenges in the area is to ensure a homogeneous spatial electric field within the tissue, enabling efficient drug delivery and full response of the tumor. Due to the heterogeneity of tumors and surrounding tissue, current density (and thus electric field distribution) is distorted in dependence on the tissue bioimpedance [14,15,16]. Compensating/mitigating the impedance of heterogeneous tissues is one of the biggest goals in the field of ECT. Additionally, minor problems also require solutions, e.g., minimization of muscle contractions, pain sensation, and oxidative damage [17,18,19].

To achieve those goals, the first works in the sub-microsecond range of pulses have appeared in the last five years [17,20,21,22]. The application of shorter pulses (nanoseconds) potentially enables a more uniform exposure due to a higher frequency component of the burst, less excitation of muscles, more flexibility in pulse parameters, and better control of delivered energy [23]. Alternatively, bipolar short pulse sequences, which boost the frequency component even further, were also studied [24,25].

The research on the bipolar cancellation effect is of utmost importance. While the application of 1–10 μs bipolar pulses resulted in the development of the technique known as H-FIRE, ensuring impedance mitigation and reduced muscle contractions, moving towards the nanosecond range was hindered by the bipolar cancellation (BPC) phenomenon [26,27]. The very first discovery of this effect was made by Pakhomov et al. [28,29]. The positive phase pulse (↑) effect is diminished by the negative phase pulse (↓), occurring in sequence one after another, regardless of doubled energy and duration As a result, the applicability of bipolar nanosecond pulses for ECT is questionable until effective solutions to overcome BPC are proposed.

In this work, we explored the bipolar cancellation effect in vitro and in vivo settings, using the B16-F10 melanoma cell line to characterize if the cancellation effect impairs Ca^2+^ electrochemotherapy. Finally, in an in vivo murine study (B16-F10 cell line, C57BL/6 mice, n = 28), we compared the efficacy of monophasic (5 kV/cm, 500 ns, n = 100) and biphasic sequences (5 kV/cm, ↑500 ns + ↓500 ns, n = 100).

## 2. Results

### 2.1. B16-F10 Cell Membrane Permeabilization

**Figure 1:**
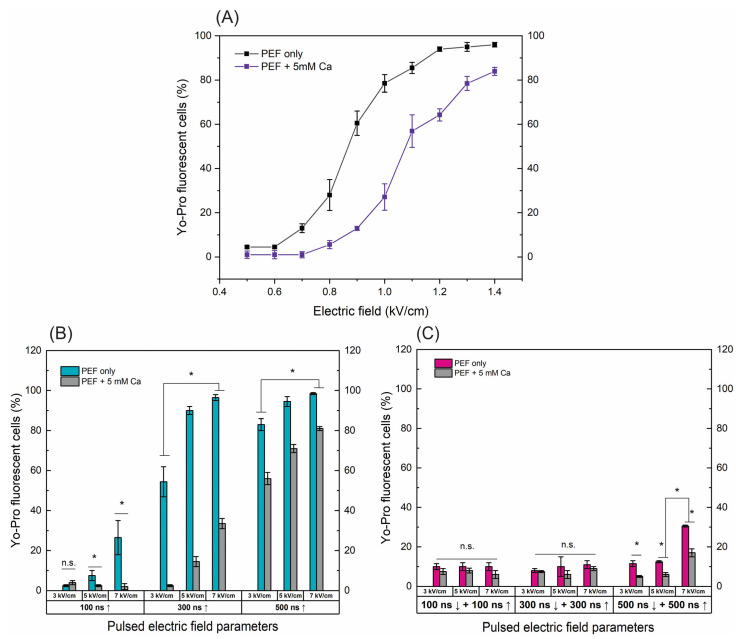
The dependence of cell permeabilization to Yo-Pro fluorescent marker on PEF protocols with and without added calcium (5 mM), where (**A**) microsecond range ESOPE sequences (100 µs × 8), (**B**) nanosecond 3–7 kV/cm sequences delivered at 1 MHz repetition frequency, duration: 100 ns, 300 ns, 500 ns, n = 100 pulses, (**C**) symmetric bipolar range 3–7 kV/cm sequences delivered at 1 MHz repetition frequency. Data presented as average ± SD. One-way ANOVA: * *p* < 0.05, n.s.—*p* > 0.05. Arrow “↑” represents positive phase of the pulse; arrow “↓” represents negative phase of the pulse.

Firstly, we have characterized electroporation efficiency using various nanosecond protocols utilizing the electrotransfer of YP following exposure to electric field pulses. Calcium ions are known to be associated with cell membrane repair and affect electroporation. Vesicular calcium ion channels and Ca^2+^ sensors gather at the sites to facilitate membrane resealing [30,31]. Therefore, the permeabilization experiments were performed with and without added calcium chloride (5 mM). The results are summarized in Figure 1.

For the ESOPE pulses (Figure 1A), an expected dose-dependent response was acquired, i.e., with an increase in electric field intensity the cell membrane permeability also increases, reaching saturated permeabilization at 1.2 kV/cm. In the case of electroporation with calcium, the efficiency of permeabilization was inhibited. At the same PEF intensity, the number of YP permeable cells decreased significantly (*p* < 0.05). E.g., in the case of the 1 kV/cm protocol, the difference is more than 40%. This tendency occurs in the whole range of tested parameters. The high permeabilization (75%+) is triggered only with the 1.3 and 1.4 kV/cm PEF protocols (Figure 1A).

Unipolar nanosecond pulses (Figure 1B) required significantly higher amplitudes to trigger high electroporation. In the case of 100 ns pulses, only 5 and 7 kV/cm PEF triggered detectable changes in the number of YP fluorescent cells; however, for effective ECT, high permeabilization is required. Increasing the duration to 300 ns improves the situation, with both 5 and 7 kV/cm being applicable for ECT (permeabilization > 90%). Similarly, a further increase in the duration to 500 ns ensured >70% permeabilization in the whole range of studied amplitudes. The addition of calcium chloride (5 mM) triggered a similar response as in the microsecond range (Figure 1A); the number of YP fluorescent cells decreased dramatically in the whole range of investigated amplitudes and durations.

Finally, the symmetric bipolar sequences were characterized using the same methodology and procedure. It can be seen (Figure 1C) that a bipolar cancellation effect is triggered. Both the ↑100 ns + ↓100 ns and ↑300 ns + ↓300 ns sequences did not trigger any significant permeabilization. In the case of ↑500 ns + ↓500 ns sequences, only 7 kV/cm protocol resulted in 30%+ permeabilization, which is significantly lower than unipolar pulses. It should be noted that the input energy of the bipolar burst is doubled when compared to the unipolar one (due to two phases of the same duration/intensity). The addition of calcium (5 mM) further reduced the efficacy of permeabilization with the highest intensity protocol, hardly achieving 20% YP fluorescent cells.

Given the promising results, our further study was limited to 500 ns protocols, which trigger a high permeabilization rate. Thus, it can potentially be used in the ECT context.

### 2.2. Effects of PEF on Cell Viability and Ca^2+^ ECT Efficiency

**Figure 2:**
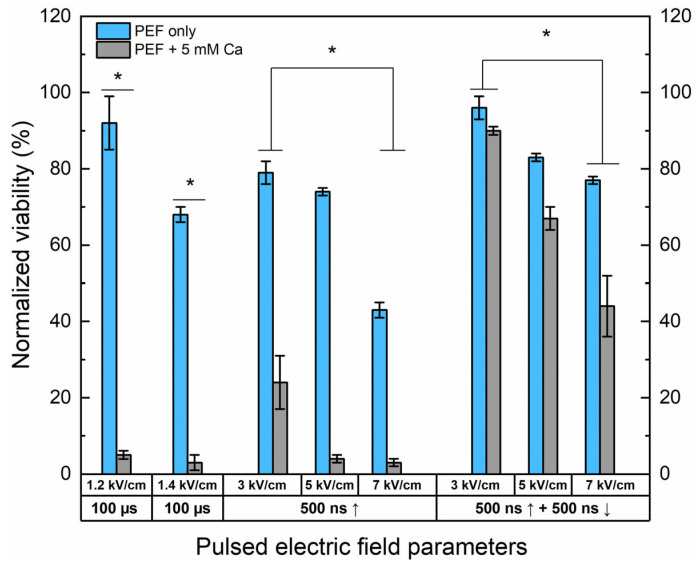
The dependence of cell viability on PEF-only and Ca^2+^ ECT protocols evaluated by metabolic cell activity assay. The 1.2/1.4 × 100 µs × 8 ESOPE sequences and 3–7 kV/cm nano-protocols have been used (500 ns, n = 100, 1 MHz). Data presented as average ± SD. One-way ANOVA: * *p* < 0.05. Arrow “↑” represents positive phase of the pulse; arrow “↓” represents negative phase of the pulse.

The effects of Ca^2+^ electrochemotherapy in vitro have been characterized. The unipolar nanosecond protocols (500 ns) have been compared with bipolar ↑500 ns + ↓500 ns sequences. The 1.2 and 1.4 kV/cm have been used as ESOPE references. The results are summarized in Figure 2.

Both 1.2 and 1.4 kV/cm ESOPE pulses induced excellent cytotoxicity when combined with calcium (5 mM). However, it should be noted that 1.4 kV/cm PEF results partly in IRE (up to 30%), while the 1.2 kV/cm protocol has no significant effect on cell viability. In the case of nanosecond pulses, 3 kV/cm Ca^2+^ ECT triggers only a partial response, with more than 20% of cells remaining viable after the treatment. Both protocols deliver a saturated cytotoxic response in the case of 5 kV/cm and 7 kV/cm sequences. Finally, the effects of bipolar sequences were of utmost interest. It can be seen that bipolar cancellation significantly hinders the efficiency of ECT, even with the highest PEF amplitude involved in the study. The cancellation phenomenon is detectable for the PEF-only treatment; the bipolar pulses are less toxic to the cells than the unipolar ones even though the energy input is 2-fold higher.

### 2.3. Thermal Influence

**Figure 3:**
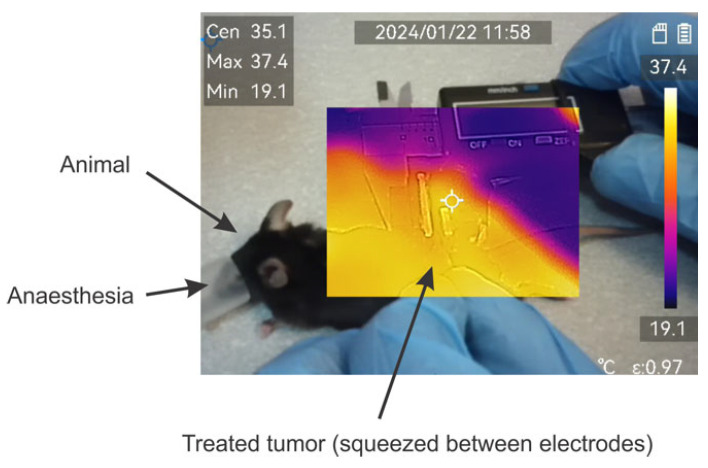
Exemplary image of thermal distribution evaluation during application of pulses. Measured using M30 thermovisor (HICMICRO, Hangzhou, China).

The effects of temperature have been evaluated using a ±2 °C accuracy M30 thermovisor (HICMICRO, Hangzhou, China). During pulse application, the temperature of the formed tumor lump was monitored throughout the burst. An exemplary photo is shown in Figure 3.

Absolutely all the protocols involved in the study did not result in detectable changes in the temperature distribution or were beyond the accuracy of the equipment used.

### 2.4. Effects of Ca^2+^ ECT In Vivo

**Figure 4:**
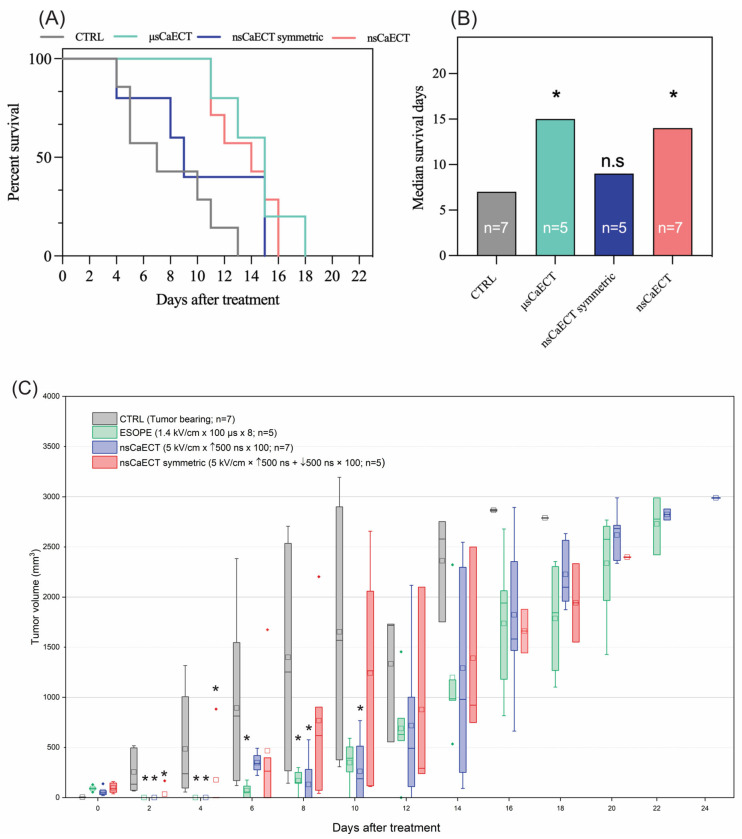
The efficiency of Ca^2+^ ECT in vivo, where (**A**) Kaplan–Meier survival curve and (**B**) median survival days, respectively. Data presented as average ± SD. Kruskal–Wallis: * *p* < 0.05, n.s. *p* > 0.05. The number of individual animals in each group is marked as “n”. (**C**) Volumetric changes in the tumors after Ca^2+^ ECT, where CTRL = untreated control; μsCaECT—1.4 kV/cm × 100 μs × 8, 1 Hz protocol; nsCaECT—5 kV/cm × 500 ns × 100, 1 MHz protocol; nsCaECT symmetric—5 kV/cm × ↑500 ns × ↓500 ns × 100, 1 Mhz; Asterisk * *p* < 0.05, Mann–Whitney U test) difference versus CTRL. Whiskers extend to maximum or minimum data values from the median of the dataset, while the top of the boxes indicates the upper quartile, and the bottom of the boxes indicates the lower quartile. The diamond indicates outliers of the data. The square represents the mean (average) of the data. The arrow “↑” represents positive phase of the pulse; arrow “↓” represents negative phase of the pulse.

For in vivo experiments (n = 24), the 1.4 kV/cm ESOPE and 5 kV/cm and 500 ns unipolar and bipolar sequences have been used. The data indicate (Figure 4A) that ESOPE pulses and unipolar nanosecond pulses trigger partial tumor response, i.e., all treated animals had prolonged survival. Monophasic nanosecond pulses were as effective (*p* < 0.004; 16 days) as the ESOPE sequence (*p* < 0.007; 18 days), resulting in tumor reduction following the treatment. Bipolar pulses did not trigger statistically significant changes compared to the untreated control (tumor-bearing mice), which agrees with the permeabilization and viability data, shown in Figure 1 and Figure 2, respectively. For the in vivo tumor volumetric changes, ESOPE pulses on days 2, 4, 6, and 8 showed statistically significant differences compared to the CTRL group (*p* < 0.05), indicating delayed tumor growth. In the CTRL group, the majority of mice had to be sacrificed by day 14 due to rapid tumor growth. For the nsCaECT group (5 kV/cm × 500 ns × 100), significant antitumor responses were observed on days 2, 4, 8, and 10. However, tumors eventually began to regrow across all groups included in the experiment. The nsCaECT symmetric group did not delay tumor growth, as tumors started to reappear as early as day 2. Compared to the CTRL group, the results were statistically insignificant (*p* > 0.05), which is in agreement with the survival data.

The control group’s median survival days (Figure 4B) were 6 days, ESOPE 15 days, unipolar nanosecond pulses 14 days, and symmetric bipolar sequences 9 days. The microsecond procedure ensured the highest survival median days. Treated mice had a median survival of 1.5–2.5 times longer than CTRL.

## 3. Discussion

The bipolar cancellation phenomenon was reported by Pakhomov et al.’s group in 2015 [32]. The phenomenon occurs in the sub-microsecond range when the second negative phase of the bipolar waveform cancels the effects of the positive phase. As a result, bipolar nsPEF is expected to be less effective than a single phase of the same pulse [33]. However, the number of works on this topic is deficient, presumably due to technological challenges, and this topic requires extensive experimental coverage. Potentially, bipolar pulses can help reduce the net charge delivered to the tissue compared to unipolar pulses, ensuring less tissue damage and less chance of inducing electrolysis and pH changes [34]. Additionally, the higher frequency component of a bipolar burst potentially allows for impedance mitigation and a reduction in muscle contractions, which were confirmed using H-FIRE methodology employing microsecond bipolar pulses [35]. This work focuses on Ca^2+^ electrochemotherapy and the challenges the bipolar cancellation introduces to the field. To the best of our knowledge, it is the first in vivo study to confirm the existence and importance of bipolar cancellation in the electrochemotherapy context.

Previously, our group has shown (in vitro) that bipolar cancellation is extreme when the repetition rate of the pulses is high (MHz range). The efficacy of electrochemotherapy (incl. Ca^2+^ ECT) is non-existent if the pulses are symmetric and delivered at a high frequency [36]. The effect of electroporation is completely mitigated, while introducing delays and/or reducing the pulses’ repetition rate minimizes the cancellation’s extent [37]. One could say that introducing delays is the solution to overcome bipolar cancellation and enable the use of bipolar nano-pulses in the ECT context. However, the efficacy of low-frequency nanosecond bursts for drug and/or gene delivery is inferior to conventional microsecond protocols (i.e., ESOPE) [38]. Reduction in the time delay between nanosecond pulses triggers a high-frequency specific polarization phenomenon, allowing the cells to stay polarized throughout the burst [39,40]. Therefore, the electrotransfer efficiency is boosted significantly, enabling effective electroporation in what was considered sub-threshold electric fields (taking into account ultra-short pulse duration) [41]. We have shown in vivo that compressing the unipolar pulses into MHz bursts improves the efficacy of ECT dramatically [42,43]. Thus, applying low-frequency nanosecond bursts for drug delivery is hardly competitive.

This work aimed to confirm if the in vitro data can be super-positioned in vivo and characterize the extent of bipolar cancellation for electrochemotherapy using actual tumors and animal models. As it was shown in Figure 4, the bipolar cancellation is a dramatic limitation of bipolar pulses to be used in the ECT context, while unipolar high-frequency bursts (two-fold lower energy input) are as effective as the ESOPE procedure. Kim et al. have shown [33] in vitro that nanosecond kinetics of membrane potential in single cells reveal the temporal summation of polarization by individual unipolar pulses applied at close to the MHz rate, leading to enhanced electroporation. In contrast, there was no summation for bipolar pulses, and increasing their repetition rate suppressed electroporation. Our results perfectly agree with the study of Kim et al., which significantly contributes to the establishment and consolidation of knowledge due to the involvement of real animals.

Regrettably, the overall efficiency of Ca^2+^ ECT in our work (even for the conventional pulses) was hardly satisfactory. While all the treated tumors responded to the treatment, there were no fully recovered animals. That fact could be attributed to the non-homogeneity of the electric field during the treatment, while the same approach delivered significantly better results when other tumor models were involved [43,44]. Therefore, we speculate that the tumor model selection influenced the non-satisfactory result. The melanoma *B16-F10* in vivo model was selected due to its representation of a significant challenge in human melanoma treatment [45,46,47,48,49,50]. Widespread melanoma with subcutaneous metastases poses an obstacle to effective treatment, given the poor immunogenicity and highly metastatic and aggressive nature of *B16-F10* tumors [51,52,53]. Although systemic therapies involving standard chemotherapy drugs have been utilized, they have yielded meager response rates [54]. In the case of subcutaneous tumors, where the majority of which were melanoma, a trend towards higher antitumor activity in non-melanoma nodules was also reported [46].

## 4. Materials and Methods

### 4.1. Cells

**Figure 5:**
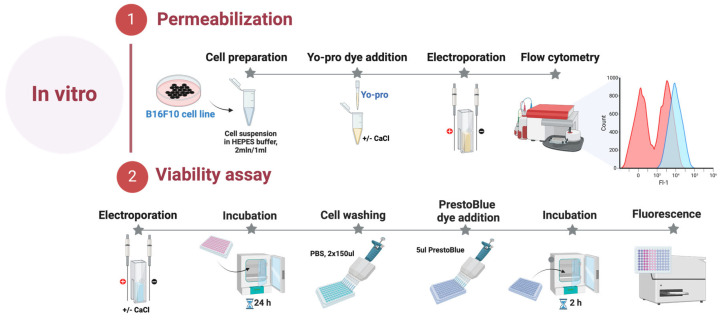
In vitro part of the experiment.

The *B16*-*F10* melanoma cells were acquired from the national collection of the State Research Institute for Innovative Medicine (Vilnius, Lithuania). B16-F10 melanoma cells were grown and maintained at 37  °C in a 5% CO_2_ Galaxy S + Incubator (RS Biotech, Lancashire, United Kingdom) in RPMI 1640 with glutamine, additionally supplemented with 100 U/mL of penicillin, 100 mg/mL of streptomycin, and 10% of fetal bovine serum (FBS) (Gibco, Thermo Fisher Scientific, Waltham, MA, United States of America). B16-F10 melanoma cells were grown in monolayers, and all experiments were performed at 80% cell culture confluency. On the experiment day, B16-F10 cells were detached using Trypsin-EDTA solution (Gibco, Thermo Fisher Scientific, Waltham, MA, United States of America), centrifuged, and resuspended in the RPMI 1640 at a 2 × 10^6^/mL concentration. All experiments were performed with mycoplasma-free cells. The cell lines were tested for mycoplasma contamination using the MycoBlue^®^ Mycoplasma Detection Kit (Vazyme, Nanjing, China). The summary is provided in Figure 5.

### 4.2. Cell Permeabilization

Electroporation-induced cell permeabilization in *B16-F10* melanoma cells was identified using the green fluorescent dye Yo-Pro1 (YP, Sigma–Aldrich, St. Louis, MO, USA). Cells suspended in the electroporation buffer (10 mM HEPES 250 mM Sucrose, 1 mM MgCl_2_) were combined with YP dye to achieve a final concentration of 1 μM with or without an additional 5 mM CaCl_2_ (Serva Feinbiochemics, Heidelberg, Germany). The 50 μL samples were placed inside the cuvette between the electrodes and treated with dedicated electroporation protocols. Afterward, the cells were transferred into a 96-well round-bottom plate (Nunc, Sigma–Aldrich, St. Louis, MO, United States of America). Following a 3 min incubation at room temperature, 150 μL of 0.9% NaCl (Chempur, Piekary Śląskie, Poland) solution was added. The control samples without treatment were used as a negative control for gate definition. After incubation, samples were measured using a BD Accuri C6 flow cytometer (BD Biosciences, San Jose, CA, United States of America), where YP (Ex. 491⁄509) fluorescence was detected in Channel FL1 (Em. 533/30 nm BPF).

### 4.3. Viability Assay

Cell viability was assessed employing PrestoBlue^®^ cell viability reagent (Thermo Fisher Scientific, Waltham, MA, United States of America) 24 h post-treatment. B16-F10 cell suspension in electroporation buffer (2 × 10^6^ cells/mL), with or without an additional 5 mM CaCl_2_, was prepared and subjected to various pulsed electric field (PEF) conditions. Subsequently, the treated cells were transferred into a 96-well flat bottom plate (TPP, Trasadingen, Switzerland). Following a 10-min incubation period, 200 μL of growth medium was added to each well, and the plate was then placed in the incubator for 24 h. On the subsequent day, the wells were gently washed with phosphate-buffered saline (PBS) (Hyclone, Lohan, UT, USA) 2 times × 150 μL (Gibco, Thermo Fisher Scientific, Waltham, MA, United States of America). After washing, 10 μL of PrestoBlue^®^ cell viability reagent was dispensed into each well. The cells were then maintained in the incubator for another 2 h, after which metabolic measurements were taken using a Synergy 2 microplate reader with Gen5 software (PN 5321002, BioTek, Shoreline, WA, United States of America). Metabolic intensity was measured at an excitation wavelength of 540/20 nm, and emission was evaluated at 620/40 nm.

### 4.4. Mice and Tumor Induction

**Figure 6:**
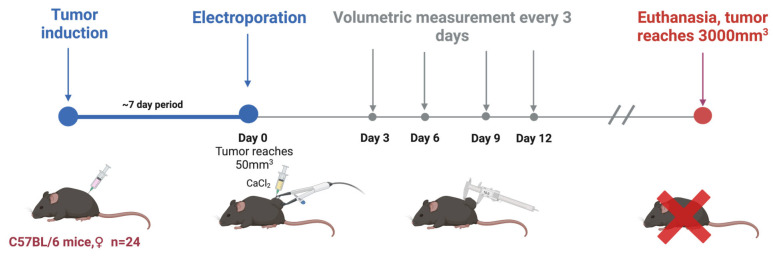
In vivo part of the experiment.

*C57BL/6* linear Mus musculus (hereinafter referred to as mice) were bred and housed at the State Research Institute Centre for Innovative Medicine’s mouse housing facility (Vilnius, Lithuania). In this study, tumors were induced in 6–8-week-old mice by subcutaneously injecting 2 × 10^6^ of *B16-F10* melanoma cells resuspended in RPMI without supplementation. Mice were randomly grouped when the tumors reached 50 mm^3^ (after 1–2 weeks; day 0). A summary is provided in Figure 6 and Table 1.

Before the electroporation treatment, the mice’s backs were shaved and then treated with an 8% aqueous solution of Na_2_S to remove hair, which was immediately rinsed with water. Prepared mice were put under anesthesia with a 3% isoflurane gas and oxygen mixture. Electroporation was conducted by compressing the tumor between flat electrodes coated with EEG and ECG ultrasound gel to ensure optimal electrical connection.

The electrodes were typically spaced 2–3 mm apart, and the charging voltage was adjusted accordingly to generate a consistent electric field within the tumor. Tumor sizes were evaluated by volumetric measurements before and every 2–3 days after the treatment.

Mice were kept until the end of the experiment (or when primary tumors had reached 3000 mm^3^), at which point they were euthanized via cervical dislocation.

### 4.5. Statistical Analysis

We employed a one-way analysis of variance (ANOVA; *p* < 0.05) to analyze the in vitro data. We conducted Tukey’s HSD multiple comparison test whenever ANOVA indicated a statistically significant result (*p* < 0.05). For in vivo data, Kaplan–Meier survival curves were generated, while significance testing (*p* < 0.05) was determined using the Kruskal–Wallis test. In vitro data processing was performed using OriginPro software (version 18.0, OriginLab, Northampton, MA, USA). In vivo data were processed using GraphPad Prism 8 (GraphPad Software, Inc., San Diego, CA, USA). All in vitro experiments were repeated at least three times, and treatment efficiency was presented as mean ± standard deviation.

## 5. Conclusions

Our study aimed to investigate how bipolar cancellation affects Ca^2+^ electrochemotherapy. We have shown that the cancellation effect is extremely strong when the pulses are closely spaced (1 MHz frequency), which results in a lack of cell membrane permeabilization and consequent failure of electrochemotherapy in vitro. At the same time, the efficacy of monophasic nanosecond pulses was comparable to the 1.4 kV/cm × 100 µs × 8 pulses sequence, resulting in tumor reduction following the treatment and prolonged survival of the animals (*p* < 0.05 versus untreated tumor-bearing control group). We report the significance of bipolar cancellation in vivo, while currently, it is indicated that symmetric bipolar pulses are hardly applicable to ECT.

## Figures and Tables

**Table 1:** Number of individuals in each group and applied treatment.

Group	Electric Field [kV/cm]	Duration	Number of Pulses [n]	Number of Individuals [n]
Control (tumor-bearing)	-	-	-	7
µsCaECT	1.4	100 μs	8	5
nsCaECT	5	500 ns	100	5
nsCaECT symmetric	5	↑500 ns + ↓500 ns	100	7

## Data Availability

The data generated in this study are available upon request from the co-responding author.
